# Growing up in a crowd: social environment shapes the offspring's early exploratory phenotype in a colonial breeding species

**DOI:** 10.1098/rsos.220839

**Published:** 2022-10-19

**Authors:** Reyes Salas, Luc Lens, Eric Stienen, Frederick Verbruggen, Wendt Müller

**Affiliations:** ^1^ Behavioural Ecology and Ecophysiology (BECO), University of Antwerp, Universiteitsplein 1, 2610 Antwerp, Belgium; ^2^ Terrestrial Ecology Unit (TEREC), Ghent University, K.L. Ledeganckstraat 35, 9000 Ghent, Belgium; ^3^ Flemish Institute for Sea Research (VLIZ), Wandelaarkaai 7, 8400 Ostend, Belgium; ^4^ Research Institute for Nature and Forest (INBO), Herman Teirlinckgebouw, Havenlaan 88, bus 73, 1000 Brussels, Belgium

**Keywords:** early-life social environment, behaviour, phenotypic plasticity, learning, colonial breeding

## Abstract

In colonial breeding species, the number of adverse social interactions during early life typically varies with breeding density. Phenotypic plasticity can help deal with this social context, by allowing offspring to adjust their behaviour. Furthermore, offspring may not be unprepared since mothers can allocate resources to their embryos that may pre-adjust them to the post-hatching conditions. Thus, we hypothesize that lesser black-backed gull chicks raised in dense breeding areas, with greater exposure to intra-specific aggression, show higher levels of anxiety and lower levels of exploration compared to chicks in low-density areas, and that this is facilitated by prenatal effects. To test this, we cross-fostered clutches within and across pre-defined high- and low-breeding density areas. We measured chicks' anxiety and exploration activity in an open-field test that included a novel and a familiar object. We found that both pre- and post-natal social environment contributed nearly equally and shaped the offspring's exploratory behaviour, but not its anxiety, in an additive way. Post-natal effects could reflect a learned avoidance of intra-specific aggression, yet identifying the pathways of the prenatal effects will require further study.

## 1. Introduction

When organisms are born, they are faced with a new, variable and challenging natal environment. Phenotypic plasticity, i.e. the ability of one genotype to express a range of different phenotypes across an environmental gradient, can help offspring to survive and optimally perform in a range of hostile environments. The latter can also be facilitated through prenatal effects, such as maternal effects whereby the mother transmits environmental information to her offspring, which serves as an important cue to offspring development [1]. When the mother correctly anticipates the post-natal environment, prenatal effects can prepare offspring for environments they are likely to encounter (i.e. anticipatory maternal effects; [2–6]). In other words, the perceived environment is translated into adaptive phenotypic variation in the offspring [7], maximizing offspring performance via developmental plasticity [8,9]. However, the adaptive significance of such maternal canalization of offspring development hinges on the predictability of the conditions after birth. If the post-natal environment deviates from the predicted environment, then the phenotype can be mismatched which will negatively affect offspring performance. The prenatal and post-natal context can therefore drive phenotypic adjustments to environmental conditions, and can become an instructive factor during development [10]. Recently, a lot of attention has been paid to the social component of the early-life environment, as the number and type of social interactions that newly born individuals have with their parents, siblings or other conspecifics (‘interacting phenotypes' *sensu* [11]) can have a major impact on a suite of behavioural traits such as exploratory behaviour, aggressive behaviour, sociability, anxiety, learning ability and personality traits [12–16].

The early-life social environment may play a particularly prominent role in colonial breeding species, since variation in breeding densities within colonies of ground-nesting species can cause strong variation in the social conditions to which offspring are exposed. For example, in colonies of *Larus* gulls, central parts with high nest densities are typically surrounded by more dispersed, isolated territories [17] where levels of aggressive interactions among individuals are generally lower [18–21]. Chicks that cross into a neighbouring territory, which is more likely to occur at higher breeding densities, may experience (possibly lethal) rates of aggression from neighbouring adults [22,23]. When establishing a breeding territory in a colony, parents therefore already set their offspring's social environment, which can have important fitness consequences for the latter. Yet, the social environment experienced by the female during laying likewise affects the deposition of maternal hormones in her eggs [24–26]. This, in turn, may pre-adjust their offspring to match post-natal (social) conditions at a given breeding density [27–29], e.g. through epigenetic effects that may result in altered hormone receptor densities, modified hormone production or morphological and muscular changes [30].

In this study, we investigated to what extent levels of anxiety and exploratory activity in the offspring of colonial breeding, semi-precocial lesser black-backed gulls vary with their early-life social environment, and whether this is modulated by prenatal effects. We hypothesized that negative (i.e. aggressive) social interactions with conspecifics, which are more common at higher breeding densities, trigger more anxious and less explorative phenotypes [31–34]. We divided our study colony into different plots of equal size, with high-density plots hosting twice as many nests as low-density ones. We then cross-fostered full clutches with the same laying dates between and within high- and low-density plots. This allowed us to separate prenatal and post-natal effects on the behavioural phenotype of the chicks, given that further adjustments of the maternally set embryonic environment are no longer possible once the eggs are laid.

Chicks were tested near fledging age using a modified open-field test. Open-field tests have been extensively used in behavioural research to study locomotor activity, exploration and anxiety [35–42]. In our version of the open-field test, we measured the behaviour of an individual after it was released into an open unfamiliar arena, which included a novel object and a familiar hiding place, to test for individual differences in exploration and anxiety. The experiment was conducted during two consecutive breeding seasons (2020 and 2021). We hypothesized that chicks reared in more dense breeding areas would be less explorative and exhibit higher levels of anxiety, and that prenatal effects would reinforce such behaviour in an additive way.

## 2. Material and methods

### 2.1. Density assessment and cross-fostering

Our study colony was located in the port of Zeebrugge, Belgium (51°20′56.2″ N 3°10′25.0″ E), and hosted 181 and 282 lesser black-backed gull breeding pairs over the studied seasons of 2020 and 2021, respectively. In order to manipulate the early-life social environment, we created eight plots of similar size (approx. 850 m^2^) by installing 50 cm high plastic mesh fences for separation. We aimed to experimentally create plots with contrasting breeding densities, i.e. plots differing in number of nests. So in 2020, we placed 15 U-shaped concrete blocks in plots that were supposed to become ‘low-density' (LD) plots and 25 blocks as shelter in those plots that should become ‘high-density' (HD) plots. The concrete blocks serve as favourable breeding spots in our population and hence attract breeding pairs. In our colony, 60% of the breeding pairs chose a territory with an artificial block, and those that did not were provided with a shelter just before chick hatching. In total, we placed 150 concrete elements in 2020, and 71 more in 2021. The concrete blocks are hence present in all plots and all chicks were familiar with them. During the breeding season of 2020, LD plots held on average 17.34 ± 3.79 nests, while HD plots held on average 35 ± 1.41 nests. In 2021, when the overall number of breeding pairs increased substantially, LD plots held on average 20.68 ± 10.41 nests versus 43 nests in the only HD plot used in 2021. Densities significantly differed between years (*χ*^2^ = 206.77, *p* < 0.001) and plots (*χ*^2^ = 9.52, *p* < 0.01).

**Figure 1.**
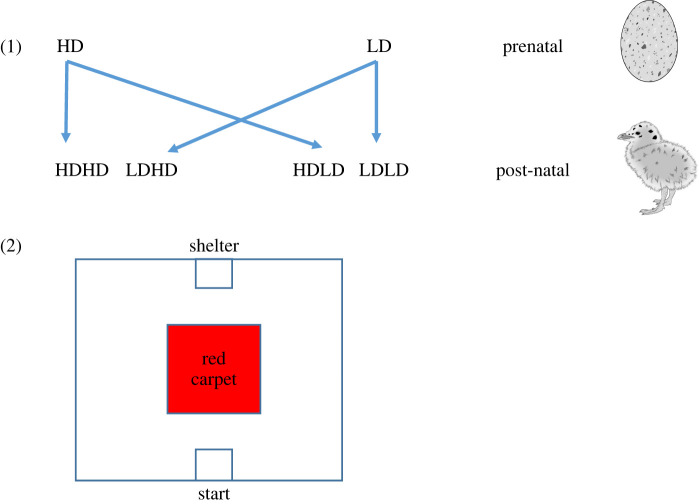
Scheme of the experimental design: (1) Cross-fostering between (=mismatching prenatal and post-natal environment) and within breeding densities (=Matching prenatal and post-natal environment) and (2) Open-field test set-up that included a familiar hiding spot, the concrete block and a red carpet as central novel object. Drawings by Reyes Reguera Rosal.

To determine laying dates and number of nests, we visited the colony three times per week from the onset of breeding onwards (± 25th of April). Typically, the peak of egg-laying is in mid-May and lasts around two weeks. The cross-fostering was carried out about one week before hatching, that is about three weeks after laying of the first egg. Not all nests were used for the experiment, as only nests where clutches could be matched for their laying dates and that were laid in a restricted time window (see below) were selected. In the breeding season of 2020, we cross-fostered a total of 108 nests within and between three LD and two HD plots. In 2021, we used three LD and one HD plot and cross-fostered 78 nests. Three of those plots (two LD and one HD) were used in both years. Clutches were cross-fostered both within the same plot and among other plots with similar or contrasting density. By cross-fostering we created four experimental groups: clutches laid in low-density (LD) plots that were cross-fostered to nests located (1) in LD plots (matching a low-density early-life environment) or (2) in high-density (HD) plots (mismatching as chicks were being raised in a more dense early-life environment); and clutches laid in HD plots that were cross-fostered to nests located (3) in LD plots (mismatching as chicks were being raised in a less dense environment) or (4) in HD areas (matching a high-density environment; figure 1).

Experimental chicks were individually labelled with numbered tape strips that were fitted around the tarsus at their first nest control after hatching (nests were checked three days a week), ensuring an accurate determination of chick origin and age. At hatch down feathers were collected for molecular sex determination [43].

### 2.2. Behavioural testing and measurements

To reduce the impact of disturbance in the colony, all tests were performed over two consecutive days and we selected chicks that were 20 ± 5 days old to control for age effects. We tested a total of 90 experimental chicks (*N*_2020_ = 46; *N*_2021_ = 44). Only one individual per nest (usually the first-hatched chick) was tested. The sample sizes among cross-foster groups and between years were as follows: *N*_LD to LD_ = 13_2020_ and 11_2021_; *N*_HD to LD_ = 12_2020_ and 13_2021_; *N*_LD to HD_ = 11_2020_ and 11_2021_ and *N*_HD to HD_ = 10_2020_ and 9_2021_.

In both years, we performed open-field tests in a 3 × 3 m arena, which had a familiar U-shaped concrete block as a familiar shelter, and a 1 × 1 m red carpet as novel object in the middle of the testing arena (figure 1). We tested each chick individually after an acclimation period of three minutes inside an opaque box that was placed in the arena at the opposite side of the shelter. After removing the box, we video recorded the chick's behaviour for 10 min. We focused on the following parameters: (i) latency to enter the shelter; (ii) time spent in the shelter; (iii) latency to enter the carpet; (iv) time spent on the carpet; and finally, (v) time spent moving and exploring (inactivity was defined to start after 5 s without movements). Each year, three testing arenas were set up in an undisturbed area nearby the colony.

After the open-field test was performed, we took morphometric measurements of chicks' head and tarsus length to the nearest 0.1 mm using a calliper, and body weight to the nearest 0.1 g using an electronic balance. We then calculated the body size (age corrected) per chick by using the residuals of a linear regression of head-bill length against age, to account for any possible effects of size. After testing and measuring, chicks were returned to their nests.

### 2.3. Data analysis

Videos were analysed using the Solomon coder software to extract the behavioural parameters of interest [44]. We then performed a Principal Component Analysis to reduce the number of behavioural traits and to account for correlations among them. The first two Principal Components (PC) had eigenvalues higher than one and were used for subsequent analyses. Specifically, to assess the effect of the early-life social environment on the chicks’ behavioural phenotype, we fitted two linear mixed models using the square-rooted PC1 (to obtain normally distributed residuals, transformed by adding the maximum negative value and a constant of 1) and PC2 respectively as response variables, and sex, year, chick hatching order (two-level factor: A or B chick), age-corrected body size, prenatal environment (two-level factor: low and high density), post-natal environment (two-level factor: low and high density), the interaction between prenatal and post-natal environment, and the interactions between both the prenatal and post-natal environment and year as fixed effects. Finally, plot ID was included as a random factor. Non-significant interactions were removed from the statistical models.

To assess which factors influenced chick size, we performed another linear mixed model with age-corrected body size as a response variable, and post-natal environment (two-level factor: low and high density), prenatal environment (two-level factor: low and high density), sex, year, the interaction between prenatal and post-natal environment, and the interactions between both the prenatal and post-natal environment and year as explanatory variables. Again, plot ID was included as random effect.

All linear mixed models were fitted using the ‘nlme' package [45] using R [46]. Normality, independence and homoscedasticity were explored by analysing model residuals. Package ‘ggplot2' was used for visualization of results [47]. Effect sizes were estimated using the ‘emmeans' [48] package, and statistical significance was set at a critical *α* level of 0.05.

## 3. Results

### 3.1. Chick size and social environment

Chicks' size (age-corrected, taken after the open-field test) was significantly larger in 2021 compared with 2020 (*χ*^2^ = 15.43, *p* < 0.001), and males were larger than females (*χ*^2^ = 77.89, *p* ≤ 0.001). However, we found no significant interactions, nor effects of the prenatal (*χ*^2^ = 1.31, *p* = 0.25) and post-natal social environment (*χ*^2^ = 0.07, *p* = 0.79) on chick size after removing non-significant interactions.

### 3.2. Principal components

PC1 explained 40.53% of the variance and was related to anxiety behaviours (shelter use duration and latency to shelter; table 1). Lower values of PC1 indicate higher anxiety levels. PC2 explained 36.72% of the variance, and was related to exploratory behaviours (time active, carpet use duration and latency to carpet; table 1). Lower values of PC2 are indicative of higher levels of exploration activity. Results of our PCA thus confirm that the open-field test measures two distinct behavioural components, namely anxiety and exploration [38,42].

**Table 1.** Output of the Principal Component Analysis showing the loadings of the two principal components with eigenvalues higher than one for each behavioural trait extracted from the open-field test.

	PC1	PC2
*loadings*		
latency to shelter	0.93	0.21
shelter use duration	−0.96	−0.09
time active	0.32	−0.71
latency to carpet	0.20	0.87
carpet use duration	0.31	−0.72

### 3.3. Behavioural phenotypes and social environment

**Figure 2.**
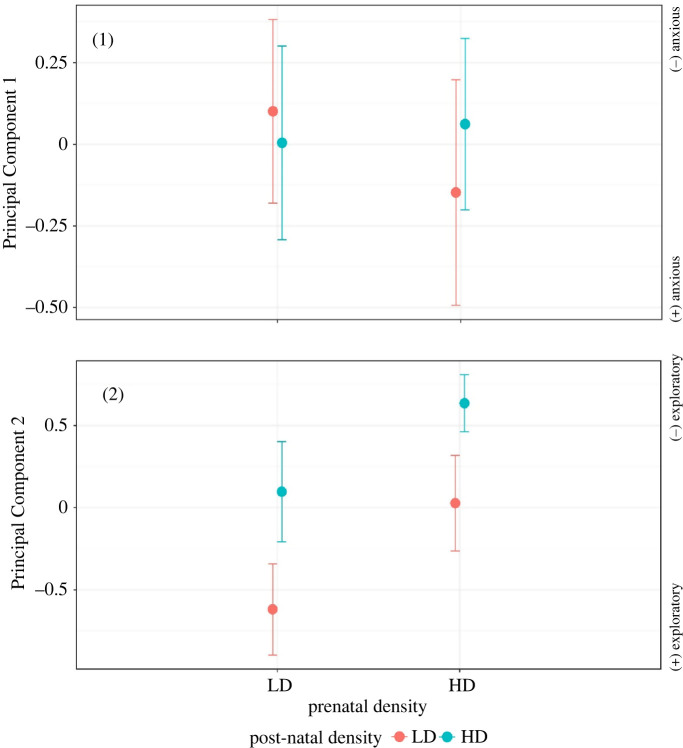
Values of (1) Principal Component 1 representing anxiety and (2) Principal Component 2 representing exploration, according to prenatal and post-natal social environments (i.e. low and high breeding densities). Dots represent the mean values, and whiskers the standard errors.

We found no significant effect of any of our explanatory variables on PC1, which reflects anxiety (table 2; electronic supplementary material, table S1). Regarding PC2, which reflects exploration activity, there was a significant effect of both the prenatal (*χ*^2^ = 3.90, *p* = 0.05) and the post-natal social environment (*χ*^2^ = 6.10, *p* = 0.01) after removing interactions in the model, as they were non-significant (table 2; electronic supplementary material, table S1). Chicks that were prenatally predetermined for LD were significantly more exploratory than the ones programmed for HD (table 2). Chicks that were raised in LD post-hatching exhibited a significantly higher exploration activity (table 2). Chicks that were raised in mismatching prenatal and post-natal conditions (LDHD and HDLD) exhibited intermediate phenotypes (figure 2). This effect was consistent in both years (table 2).

**Table 2.** Full-linear mixed models excluding non-significant interactions (1) testing the effect of sex, year, chick size, prenatal and post-natal conditions, and the interaction between the prenatal and post-natal conditions on the Principal Component 1 (anxiety). A similar model (2) was fitted to investigate the effects on Principal Component 2 (exploration activity). The reference levels of comparison were: LD for prenatal and post-natal densities, 2020 for year, females for sex and a chick for chick hatching order.

	coefficient	s.e.	*χ*^2^	d.f.	*p*-values	*effect size ± s.e.*
*1. Principal Component 1*						
prenatal density	−0.05	0.08	0.40	1	0.53	*0.13 ± 0.21*
post-natal density	0.01	0.10	0.02	1	0.89	−*0.04 ± 0.27*
year	−0.07	0.09	0.63	1	0.43	*0.19 ± 0.23*
sex	0.00	0.11	0.00	1	0.98	−*0.01 ± 0.30*
chick size	0.02	0.01	2.06	1	0.15	
chick order	0.01	0.09	0.00	1	0.95	−*0.01 ± 0.23*
*2. Principal Component 2*						
prenatal density	0.55	0.28	3.90	1	0.05	−*0.42 ± 0.21*
post-natal density	0.70	0.28	6.10	1	0.01	−*0.54 ± 0.22*
year	0.24	0.30	0.67	1	0.41	*0.05 ± 0.07*
sex	0.47	0.39	1.46	1	0.23	−*0.00 ± 0.09*
chick size	−0.00	0.05	0.00	1	0.98	
chick order	0.19	0.30	0.40	1	0.53	−*0.00 ± 0.07*

## 4. Discussion

An important component of the early-life environment that most animal species must cope with is the social environment. Animals might hence—from the moment they are born—have to adapt to the social context via phenotypic plasticity to increase their performance and to reduce their chances of mortality. Here we indeed show that the behavioural phenotype of lesser black-backed gull chicks is significantly altered in relation to their post-hatching social environment, consistently so over two consecutive years. Furthermore, this phenotypic effect was supported by the contribution of prenatal effects, as such that the prenatal effects had an additive effect on the offspring's phenotype. Below we discuss our results against the background of developmental plasticity, which might play an important role in shaping behavioural differences, possibly with long-lasting consequences.

### 4.1. Post-natal effects

The adult breeding territory of colonial breeding lesser black-backed gulls determines the offspring's early-life social environment, and we hypothesized that it affects the expression of their behaviour, as it is sensitive to and modulated by environmental conditions. Thus, growing up in territories in more dense areas should trigger more anxious and less explorative offspring phenotypes, as they experience more aggressive interactions from neighbouring breeding pairs and/or their chicks [18,20,22,23]. Consistent with this hypothesis, the post-natal breeding density to which a chick was exposed explained a significant amount of variation in its exploration activity, with chicks being raised in HD areas showing reduced exploration activity. Lesser black-backed gulls are semi-precocial, and their chicks start moving around when about 5–7 days old. As we tested the chicks when they were about three weeks old, it is likely that chicks might have learned to reduce their exploration activity through social experience. That is in particular by aversive feedback learning, as they might have encountered aggressive interactions with neighbouring birds when moving beyond their own territory borders. Chicks raised in HD areas would benefit most strongly from being less explorative and hence from avoiding conflicts, as their territories are probably smaller and encounters with neighbours more likely. Unfortunately, we do not have reliable estimates of chick mortality in our colony, and we obviously do not have the behavioural profile of chicks that died before the testing, while this could have provided relevant information on the adaptive significance of a given behavioural phenotype in a certain social context.

Furthermore, the observed behavioural differences among chicks raised in low and high breeding plots could also be related to the behaviour of their foster parents. The amount and quality of parental care during development have been correlated with offspring behaviour in general [49–54], and here specifically one parental behaviour, the parental presence at the nest, could be of importance. Gull chicks tend to stay in close proximity to their parents, and via alarm calls parents may force their chicks to remain quiet and hide in safe spots (here: the concrete nest blocks) [55,56]. This would result in an enhanced protection of the offspring from intra-specific aggression through reduced exploration activity, again likely through learning even though here chicks might not have experienced aggression.

Intriguingly, we did not find effects of the post-hatching social environment on chick anxiety. The anxiety of the chicks raised in HD areas was comparable to that of chicks raised in LD areas. At present, we can only speculate that anxiety could be a less plastic trait. Possibly because anxiety is a key trait so that all chicks in the colony show similar intrinsic tendencies to hide when left alone by their parents and in potential danger, as it might enhance survival. However, we do observe among individual variation in anxiety, yet we could not identify the underlying drivers (see also below), we only could rule out an effect of the post-natal social environment.

### 4.2. Prenatal effects

We further hypothesized that chick behaviour might be pre-adjusted by the prenatal (social) conditions. This hypothesis was also supported by our data as prenatal breeding density significantly predicted the exploration activity by the offspring, explaining about 50% of the variation. More specifically, chicks born from eggs laid by females breeding in HD plots, but raised in LD ones, showed similar exploration activity levels to chicks born from eggs laid by females in LD plots but raised in HD ones. Moreover, their level of exploration activity was intermediate to that of chicks raised from clutches that had been cross-fostered within high- or low-density plots. We did not find any significant interaction between pre- and post-natal environments on offspring's behavioural phenotype, but only two significant main effects. Thus, pre- and post-natal effects were about equal strength and shaped offspring phenotype in a similar way, i.e. they were additive. This suggests that offspring may benefit from the prenatal programming when the post-hatching conditions are similar to the prenatal conditions, thus in a matching environment.

From a proximate perspective, maternal yolk androgens constitute a prime candidate for pre-adjusting offspring behaviour to the social conditions experienced after hatching [28]. Breeding density as experienced by the mother when laying her eggs (here referred to as the social environment) has earlier been shown to alter maternal androgen deposition into the egg yolk [26,57–60]. Maternal yolk androgens are known to affect a variety of offspring traits [28,61], such as begging, exploratory and territorial behaviour [29,62,63]. The latter could hence serve as functional explanations for the fact that yolk androgen levels often covary with breeding densities. However, we found a lower exploration activity in high breeding densities, where yolk androgens levels are supposed to be high, which contrasts the still limited current evidence [62–64].

Other maternally derived hormones might be involved, such as corticosterone [65] which is involved in stress responses, and high breeding density environments could affect maternal stress levels. However, their transmission to the egg by the mother is still debated [66]. If deposited by the female, they should induce greater levels of fearfulness and anxiety in the offspring ([67,68], but see [69]), yet we did not find a prenatal effect on anxiety. Finally, the prenatal effects might also reflect genetic differences in exploratory behaviour (e.g. [70–74]). While a low exploration activity is likely adaptive for the offspring in high-density areas, it remains puzzling how this could be adaptive in adults.

Other non-mutually exclusive prenatal mechanisms with potential effects on offspring behaviour include more disrupted incubation patterns in HD areas due to more frequent interactions with neighbouring pairs [75,76], or vocal cues (i.e. alarm calls) experienced during the embryonic phase, which would be more present in HD areas [77]. We could have underestimated the role of prenatal cues, as some clutches were already cross-fostered days before hatching (cross-fostering of all clutches was synchronized to avoid disturbance after chicks had hatched). However, there were no differences in the average time (i.e. days after cross-fostering) clutches spent in their foster plots relative to their hatching day and according to cross-foster category (*χ*^2^ = 2.33, *p* = 0.51). Thus, further studies are necessary to unravel the proximate mechanisms underlying prenatal effects.

## 5. Conclusion

We provide key evidence that the early-life social environment plays a fundamental role in shaping the offspring's behavioural phenotype in our study species. Prenatal effects, i.e. maternal effects, and post-natal effects, i.e. learning through social interactions or parental care, contribute equally and uni-directionally to variation in exploration activity. The fact that both prenatal and post-natal stages impact on offspring phenotype implies that the timeframe during which developmental plasticity and adjustment to the prevailing social environmental context can occur, is extensive. Understanding the adaptive significance of the observed patterns will ultimately require a better understanding of the mechanisms, i.e. the contribution of maternal effects and genes to offspring performance, as well as of potentially long-lasting effects during adulthood.

## Data Availability

The data are provided in electronic supplementary material [78].
